# Putting Ethnobotany into Practice: In Vitro Antioxidant Potential and Impact on Rat Gastric Smooth Muscle Reactivity of Aqueous Extracts of *Marrubium friwaldskyanum* Boiss. and *Marrubium peregrinum* L.

**DOI:** 10.3390/life15060948

**Published:** 2025-06-12

**Authors:** Mariya Choneva, Anelia Bivolarska, Donika Gyuzeleva, Valentin Turiyski, Plamen Stoyanov, Tsvetelina Mladenova, Krasimir Todorov, Rumen Mladenov, Natalia Prissadova, Raina Ardasheva, Viktor Yotov, Petko Denev, Aleksandrina Topalova-Shishmanova, Stoyan Bivolarski, Ivica Dimov

**Affiliations:** 1 Department of Medical Biochemistry, Faculty of Pharmacy, Medical University of Plovdiv, 15A Vasil Aprilov Blvd., 4002 Plovdiv, Bulgaria; anelia.bivolarska@mu-plovdiv.bg (A.B.); ivica.dimov@mu-plovdiv.bg (I.D.); 2 Department of Botany and Biological Education, Faculty of Biology, University of Plovdiv “Paisii Hilendarski”, 24 Tsar Assen Str., 4000 Plovdiv, Bulgaria; dgyuzeleva@uni-plovdiv.bg (D.G.); pstoyanov@uni-plovdiv.bg (P.S.); cmladenova@uni-plovdiv.bg (T.M.); ktodorov@uni-plovdiv.bg (K.T.); rum.mlad@uni-plovdiv.bg (R.M.); 3 Department of Medical Physics and Biophysics, Faculty of Pharmacy, Medical University of Plovdiv, 4002 Plovdiv, Bulgaria; valentin.turiyski@mu-plovdiv.bg (V.T.); natalia.prisadova@mu-plovdiv.bg (N.P.); rayna.ardasheva@mu-plovdiv.bg (R.A.); viktor.yotov@mu-plovdiv.bg (V.Y.); 4 Department of Bioorganic Chemistry, Faculty of Pharmacy, Medical University of Plovdiv, 15A Vasil Aprilov Blvd., 4002 Plovdiv, Bulgaria; 5 Laboratory of Biologically Active Substances, Institute of Organic Chemistry with Centre of Phytochemistry, Bulgarian Academy of Sciences, 139 Ruski Blvd., 4000 Plovdiv, Bulgaria; petko.denev@orgchm.bas.bg; 6 Department of Otorhinolaryngology, Faculty of Medicine, Medical University of Plovdiv, 15A Vasil Aprilov Blvd., 4002 Plovdiv, Bulgaria; aleksandrina.topalova@mu-plovdiv.bg; 7 Faculty of Biology, University of Plovdiv “Paisii Hilendarski”, 24 Tsar Assen Str., 4000 Plovdiv, Bulgaria; stoyanbiv@gmail.com

**Keywords:** *Marrubium* genus, *M. friwaldskyanum*, *M. peregrinum*, plant extracts, antioxidant potential, smooth muscle reactivity

## Abstract

The genus *Marrubium* (Lamiaceae) is widely used in traditional medicine. While some representatives of the genus have been well investigated, the biological activity of others remains largely unknown. The aim of the current study was to assess the in vitro antioxidant potential and the effect on the reactivity of isolated rat gastric smooth muscles (SM) of aqueous extracts of *Marrubium friwaldskyanum* inflorescences, stems and leaves, and *Marrubium peregrinum* as a whole herb. The antioxidant activity was analyzed through multiple spectrophotometric and fluorometric assays. The effect on SM reactivity was determined by the treatment of excised gastric muscles of 10 male Whistar rats with the plant extracts alone or successive to 1,1-dimethyl-4-diphenylacetoxypiperidinium iodide, ketanserin, verapamil, and acetylcholine. According to the obtained results, the *M. friwaldskyanum* leaf extract exhibited the greatest antioxidant potential, followed by the *M. peregrinum* one. The SM reactivity analysis revealed that the treatment with all four extracts induced a dose-dependent contractile response with predominant cholinergic character. However, activation of serotoninergic and/or dopaminergic pathways was also observed. Furthermore, when applied after verapamil, the extracts showed a SM relaxant effect. The discovered biological activity will serve as a basis for future analyses through which the therapeutic effect of the plants will be investigated.

## 1. Introduction

Medicinal plants of wild origin have been used in folk medicine since prehistoric times. Traditional ethnobotanical knowledge has sustained the usage of herbal teas and infusion beverages as natural remedies for the treatment of various diseases and ailments to the present day [1,2]. The number of plant species used in traditional medicine is said to be 70,000, with as much as 15% of these species grown worldwide having been investigated for their biological activities [3].

The genus *Marrubium* (Lamiaceae) comprises between 40 and 50 species spread throughout Europe, South America, North Africa and Asia [4,5]. The therapeutic potential of the genus has been well investigated and some of its representatives have been widely used in traditional medicine for the treatment of respiratory diseases, gastrointestinal disorders, inflammation and pain [6,7]. Among the vast range of biological activities these plants exhibit are antioxidant, anti-inflammatory, antimicrobial, antidiabetic and anti-hypertensive. Most of these activities could be ascribed to the phytochemical profile of the *Marrubium* species, which includes a variety of polyphenols, diterpenes, and sterols [5,6].

Four *Marrubium* species are native to Bulgaria—*M. vulgare*, *M. parviflorum* Fisch. et. Mey., *M. peregrinum* L. and *M. friwaldskyanum* Boiss. [8,9]. *M. friwaldskyanum* is a perennial herbaceous plant and a Bulgarian endemite with narrow distribution, limited to the Rhodope Mountain and the Thracian Plain. The Red Data Book of the Republic of Bulgaria classifies it as critically endangered [10]. The vast resemblance in the morphology of *M. friwaldskyanum* with the most studied species of the genus—*M. vulgare*, a US bestseller herbal supplement for 2018, has led scientists to believe that the exploration of this endemic Bulgarian species is worth the effort [10,11]. *M. peregrinum* is a perennial herbaceous plant, listed in the Medicinal Plants Act (2000) [9].

Our previous research on *M. friwaldskyanum* and *M. peregrinum* involved untargeted metabolome profiling, which revealed the presence of metabolites such as flavonoids, phenolic acids, sesquiterpenes, and triterpenoids. Furthermore, there was a notable difference in the secondary metabolite content on a tissue level in *M. friwaldskyanum* [12]. Recently, we also explored the antitumor and antibacterial properties of the species [13].

Based on the high content of compounds with antioxidative properties such as flavonoids and phenolic acids present in both *Marrubium* species, we decided to further expand our studies on the biological activity of the plants by investigating their antioxidant potential. Significant antioxidant activity has been reported for several *Marrubium* species, including *M. vulgare* [14], *M. sivasense* Aytaç, Akgül & Ekici [15], *M. globosum* Montbret and Aucher ex Benth. [16], *M. crassidens* [17], and *M. deserti* de Noé [18], as well as the endemic species *M. trachyticum* Boiss. [19], *M. rotundifolium* Boiss. [20], and *M. cordatum* Nabelek [21].

A few studies on the antioxidant potential of *M. peregrinum* exist; however, they involve the essential oil [22] or a methanolic leaf extract [23]. A study also reports on the antioxidant activity of a methanolic herbal extract of *M. friwaldskyanum*, cultivated in Poland [5].

A major application of some representatives of genus *Marrubium* in traditional medicine is for the treatment of gastrointestinal diseases [11]. *M. vulgare* has been reported to exhibit significant antispasmodic [24] and gastroprotective effect [7]. Antispasmodic effect has also been discovered for *M. globosum* ssp. *libanoticum* [25]. No previous evaluation of the effect of *M. friwaldskyanum* and *M. peregrinum* on the contractility of gastric smooth muscles was found in our literature preview.

The considerable insight into the primary and secondary metabolites synthesized by both plants, provided by the previously conducted metabolome profiling [12] as well as the existence of some research gaps on their biological activity, presented an opportunity for the current research to address the in vitro antioxidant potential and the effect of the plants on the reactivity of isolated rat gastric smooth muscles.

## 2. Materials and Methods

### 2.1. Drugs and Chemicals

All chemicals, including 2,2-diphenyl-1-picrylhydrazyl (DPPH), 2,4,6-tri(2-pyridy)-s-triazine (TPTZ), 2,2-azobis-(2-amidinopropane) dihydrochloride (AAPH), 6-hydroxy-2,5,7,8-tetramethylchroman-2-carboxylic acid (Trolox), Folin–Ciocalteu’s phenol reagent, fluorescein disodium salt, gallic acid, quercetin-3-rutinoside, 1,1-dimethyl-4-diphenylacetoxypiperidinium iodide (4DAMP), ketanserin, verapamil, acetylcholine (ACh), serotonin (5-HT), and solvent methanol, were purchased from Sigma-Aldrich (Steinheim, Germany).

### 2.2. Plant Material and Extract Preparation

**Figure 1:**
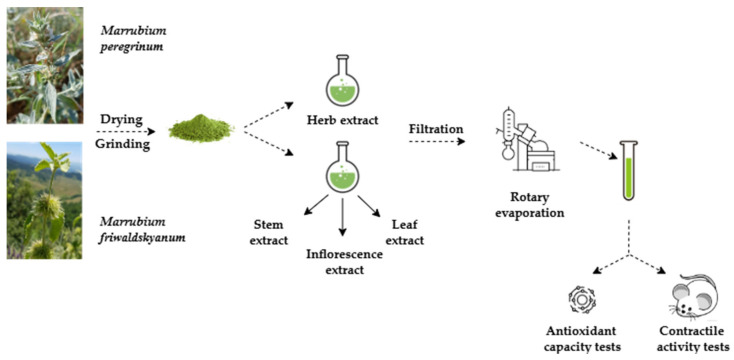
Experimental phases.

The aerial parts of *M. friwaldskyanum* and *M. peregrinum* were collected in the summer (August–September) of 2021 in their natural habitat in the Rhodope Mountains. The specimen numbers obtained upon deposition at the Herbarium of the Agricultural University, Plovdiv, Bulgaria were reported previously [12]. The schematic representation of the experimental phases is presented in Figure 1. Excised stems, leaves, and inflorescences of *M. friwaldskyanum,* as well as the whole herb of *M. peregrinum,* were dried at room temperature in a shaded area and were afterwords finely ground in a mechanical grinder (GRINDOMIX GM200, RETSCH GmbH, Haan, Germany) to a powder size of less than 400 μm. Due to the small size of *M. peregrinum*, and with a view of obtaining a sufficient for all present and previous experiments amount of the plant extract, we refrained from dividing the plant into its anatomical parts. The obtained powdered samples were stored in a dark place in tightly sealed vials until the conduction of the analysis.

The following phytochemical extracts were prepared:

1. w.e.—water extract from *M. friwaldskyanum* inflorescences;
2. w.e.—water extract from *M. friwaldskyanum* stems;
3. w.e.—water extract from *M. friwaldskyanum* leaves;
4. w.e.—water extract from *M. peregrinum* herb.

The extraction process was performed with 10 g of ground plant material that was extracted with double-distilled water (1:10 *w*/*v*). The extraction was conducted at room temperature in a flask protected by light by continuous stirring for 24 h and a triplicate ultrasonic extraction (three 15 min cycles at 30 °C). The extraction was followed by centrifugation at 6000× *g* for 15 min and filtration of the obtained supernatant with Whatman No. 1 filter paper. The same procedure was repeated twice more on the plant material that remained. The three extracts were afterwords combined, and the solvent was evaporated to complete dryness in a rotary evaporator (Heidolph, Schwabach, Germany) under reduced pressure at 50 °C. Post evaporation, the extraction yield for each extract was calculated using the following equation:

Y = [(Weight of the dry extract (g)/Weight of the ground plant (g)) × 100]

where Y is the extraction yield, % (*w*/*w*).

### 2.3. Determination of the Antioxidant Content and Antioxidant Capacity

#### 2.3.1. Total Polyphenol (TP) and Total Flavonoid (TF) Content

The TP was determined in accordance with the method of Singleton and Rossi using Folin–Ciocalteu reagent and gallic acid (GA) as a standard [26]. The results are expressed as mg gallic acid equivalents (GAE) per 100 g dry weight (DW).

The TF content was measured with AlCl_3_ reagent in accordance with the method of Chang et al. [27]. Quercetin dihydrate (10–200 mg/L) was used for the construction of a standard calibration curve and the results are expressed as mg quercetin equivalents (QE) per 100 g DW.

#### 2.3.2. Determination of the Radical Scavenging Activity by DPPH Method

The determination of DPPH radical scavenging activity (RSA) of the tested extracts followed the method of Brand-Williams et al. with certain modifications [28]. Fifty µL of the extract were added to 1.7 mL of 80 µM DPPH. The samples were kept in the dark for 20 min and their absorbance at 517 nm was measured against a control containing 50 µL of 99% methanol and 1.7 mL of 80 µM DPPH.

The activity of extraction of radicals of the studied phytochemical extracts was determined by the following equation:

% RSA = [(Abs_control_ − Abs_sample_/Abs_control_) × 100] (1)

#### 2.3.3. Determination of the Total Antioxidant Activity of the Tested Extracts

The Ferric-reducing antioxidant power (FRAP) method of Benzie and Strain, with certain modifications, was used for the determination of the total antioxidant activity [29]. The FRAP reagent was prepared directly before use by mixing 100 mL of 300 mM sodium acetate buffer (pH 3.6), 10 mL of TPTZ solution and 10 mL of FeCl_3_ × 6 H_2_O solution. For the FRAP assay, 2.85 mL of the FRAP reagent and 0.15 mL of the tested extracts were mixed and incubated in the dark at 37 °C for 30 min. The absorbance of the mixture was measured at 593 nm against a standard calibration curve of Trolox (0–1.5 mM/mL). The results are expressed as µM Trolox equivalent per g of DW.

All spectrophotometric measurements were performed on an Evolution 300 UV-Vis spectrophotometer (Thermo Fisher Scientific, Waltham, MA, USA).

#### 2.3.4. Oxygen Radical Absorbance Capacity (ORAC) Method

The ORAC assay was performed according to Ou et al. [30]. A FLUOstar OPTIMA fluorimeter (BMG LABTECH, Offenburg, Germany) with an excitation wavelength of 485 nm and emission wavelength of 520 nm was used. The results were compared to a standard calibration curve of Trolox (6.25, 12.5, 25, 50, and 100 μM) and are expressed as µM Trolox equivalent per g sample.

#### 2.3.5. Hydroxyl Radical Averting Capacity (HORAC) Method

The HORAC method was performed according to Ou et al. [31]. A FLUOstar OPTIMA fluorimeter (BMG LABTECH, Offenburg, Germany) with an excitation wavelength of 485 nm and emission wavelength of 520 nm was used for the measurements. GA solutions (100, 200, 400, 500, and 600 µM) were used for the construction of a standard curve. The results are expressed as µM GA equivalent per g sample.

### 2.4. Experimental Animals

All experimental protocols complied with the rules and regulations for the use of laboratory animals. The study obtained a permit form the Bulgarian agency for Food Safety (BAFS Resolution № 327/9 December 2021) and the Ethics Committee of the Medical University of Plovdiv (Protocol № 5/17 June 2022) and followed the guidelines of the European Directive—22.09.2010 (210/63/EU) for work with experimental animals.

The objects of the study were 10 mature male Wistar rats, weighing between 190 and 220 g, that were sourced from the vivarium of the Medical University of Plovdiv. The animals were kept under standard living conditions: temperature of 23 ± 1 °C, 12/12 h light–dark cycle, relative humidity of 45%, free access to food and water.

### 2.5. Assessment of the Contractile Activity and Reactivity of Isolated Stomach Smooth Muscles (SM)

#### 2.5.1. Rat Preparations

The in vitro studies aimed for the elucidation of the changes in the mechanical activity of stomach SM upon treatment with the studied plant extracts and other biologically active substances.

At the end of the experimental period the animals were decapitated, and, immediately after that, stomach SM preparations (corpus) were excised in situ, separating only the muscle tissue without mucosa. During preparation, the SM tissue was continuously washed with a cold preparation solution that was aerated in advance. The solution contained 120 mM NaCl, 5.9 mM KCl and 2.5 mM CaCl_2_ in a ratio 27.2/1.1/1. The circular rat stomach preparations used for the registration of the contractile activity were 15–18 mm long and 1–1.1 mm wide. The preparations were fixed in a tissue bath with Krebs solution (pH = 7.4, t^o^ = 37 °C) with the following contents (mM): NaCl—120; KCl—5.9; CaCl_2_—2.5; MgCl_2_—1.2; NaH_2_PO_4_—1.2; NaHCO_3_—15.4 and glucose—11.5. All chemicals used for the solution were purchased from Merck (Darmstadt, Germany). The Krebs solution was constantly aerated with a 19/1 (*v*/*v*) O_2_/CO_2_ mixture. The pH value of the solution was measured by HANNA HI5521-02 (Hanna instruments, Washington, DC, USA).

#### 2.5.2. Contractile Activity Measurement

The registration of mechanical activity was conducted following an isometric method. The latter allows for a quantitative evaluation of the reactions of contraction in mN. The SM preparations were fixed at one end to a glass holder that rendered them immobile, while their other end was connected by surgical thread to Swema strain gauges (Stockholm, Sweden), that convert the mechanical deformation, caused by the contractions, into a magnitude proportional electrical signal. The signal was amplified by a K. Teaser—D 486 (Karben, Germany). The mechanical activity was recorded on a paper tape by a Linseis recorder (Selb, Germany).

The initial mechanical load for the preparations achieved by stretching corresponds to a tensile force of 10 mN. The adaptation period for the establishment of a baseline tone level and the regular spontaneous contractile activity (SCA) was 60 min (during this period the solution was replaced with a fresh one 2–3 times). The changes in the SCA and tone, caused by altered conditions or by the influence of different substances, were registered against the corresponding baseline value.

Exposure to the studied substances was ensured by the addition of a precise volume of a concentrated solution of the respective compound, that was necessary to achieve the desired concentration in the tissue bath. For the extract studies, consecutive applications were made (e.g., mediator + extract or blocker + extract). The volume did not exceed 1/10 of the volume of the solution in the tissue bath. The vitality of the SM tissue was tested by exposure to 1 × 10^−6^ M ACh at the beginning and end of each experiment.

The following biologically active substances were used for the treatment: 4DAMP—a selective M_3_ antagonist of the muscarinic ACh receptors (mAChR); ketanserin—a serotonin receptor (5-HT_2_) antagonist; verapamil—a L-type calcium channel blocker used for the treatment of hypertension, and ACh.

### 2.6. Statistical Analysis

The statistical analyses were conducted with SPSS, version 17.0 (IBM, Chicago, IL, USA). All experiments that involved plant extracts were conducted in triplicates and are presented as mean values ± standard deviation (SD). The one-way ANOVA and Tukey’s post hoc test was used for the intergroup comparisons. The software Excel (Office 2016, Microsoft, Redmond, WA, USA) was used for the graphical representation of the results. The statistical significance was set at *p* ≤ 0.05.

## 3. Results

### 3.1. Extraction Yield

Table 1 demonstrates the findings for the extraction yield for the four different extracts. As can be seen from the results, the extraction yield was higher in the aqueous *M. friwaldskyanum* leaf extract compared to the other anatomical part extracts and the *M. peregrinum* herb extract.

### 3.2. Antioxidant Content and Antioxidant Activity

#### 3.2.1. Total Polyphenols and Flavonoids

The data for TP and TF content of the studied extracts are presented in Table 2. As evident from the results, the *M. friwaldskyanum* leaf extract had the highest amount of both polyphenols and flavonoids compared to the other anatomical parts and the *M. peregrinum* extract (*p* ≤ 0.001). The *M. friwaldskyanum* stem extract and *M. peregrinum* herb extract had similar antioxidant content, while the *M. friwaldskyanum* inflorescence extract showed the lowest TP and TF content (*p* ≤ 0.001 compared to the rest of the extracts).

#### 3.2.2. Antioxidant Activity

The antioxidant capacity of the studied plant extracts was assessed through an array of spectrophotometric and fluorometric methods. The results are presented in Table 3. According to the comparative analysis, the *M. friwalsdskyanum* leaf extract exhibited greater antioxidant capacity than the rest in the ORAC and HORAC tests (*p* ≤ 0.001). Similar results were obtained in the DPPH assay for 3 w.e. (*p* ≤ 0.01 vs. 1 w.e. and 2 w.e.) and 4 w.e. (*p* ≤ 0.01 vs. 1 w.e. and *p* ≤ 0.05 vs. 2 w.e.). In accordance with the TP and TF content, the highest antioxidant capacity was observed for the leaf aqueous extract, followed by the *M. peregrinum* herb extract, which underlines their more pronounced biological activity.

### 3.3. Effect of the Plant Extracts on the Contractility of Isolated Rat Gastric Smooth Muscles

#### 3.3.1. *M. friwaldskyanum* Inflorescence Extract

**Figure 2:**
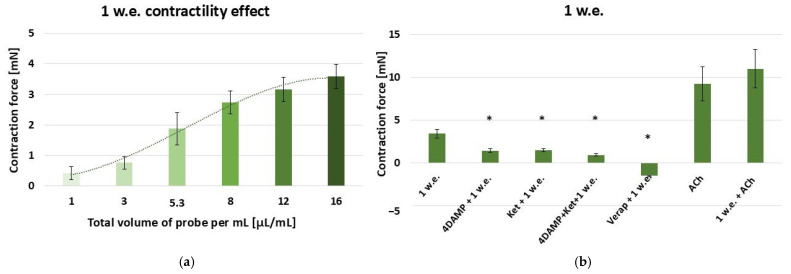
(**a**) A dose-dependent effect of *M. friwaldskyanum* inflorescence extract (1 w.e.) on the contractility of isolated gastric SM samples (n = 6); (**b**) Induced mechanical responses of gastric SM samples (n = 6) by different biologically active substances—alone and in combination with 1 w.e. Effective doses: 1 w.e.—10 µL/mL; 4DAMP—1 × 10^−6^ M; ketanserin—1 × 10^−6^ M; verapamil—1 × 10^−5^ M; Ach—1 × 10^−6^ M. The data is presented as Mean ± SD. The symbol * indicates significant differences versus the control group.

According to the obtained results, the aqueous inflorescence extract provokes a dose-dependent contractile reaction of the gastric SM in the following range: 10–160 µL (effective work dose range: 1–16 µL/mL) [Figure 2a].

Further exposure was conducted with an effective dose of 10 µL/mL (control), which alone initiated a response of 3.43 ± 0.5 mN. The data are presented in Figure 2b. In comparison, when applied approximately 5 min after 1 × 10^−6^ M 4DAMP, 1 w.e. led to a contraction with force of 1.46 ± 0.21 mN, with the response being significantly reduced against the control (*p* ≤ 0.05). Similarly, when applied after 1 × 10^−6^ M ketanserin, the extract led to a contraction of 1.47 ± 0.18 mN, which again was significantly lower than the control one (*p* ≤ 0.05). The mixture of 1 × 10^−6^ M 4DAMP, 1 × 10^−6^ M ketanserin and 10 µL/mL of the aqueous inflorescence extract induced a response of 0.94 ± 0.15 mN. Therefore, the contraction force was significantly reduced compared to the control one (*p* ≤ 0.05). Applied approximately 5 min after verapamil (1 × 10^−5^ M), 1 w.e. provoked relaxation of −1.5 ± 0.1 mN, which was significantly different compared to the control reaction (*p* ≤ 0.05). The application of ACh in concentration 1 × 10^−6^ M induced a mean contraction of 9.25 ± 1.98 mN. When combined with 1 w.e., the obtained response was 11 ± 2.23 mN. No significant difference compared to the sole effect of ACh was observed.

#### 3.3.2. *M. friwaldskyanum* Stem Extract

**Figure 3:**
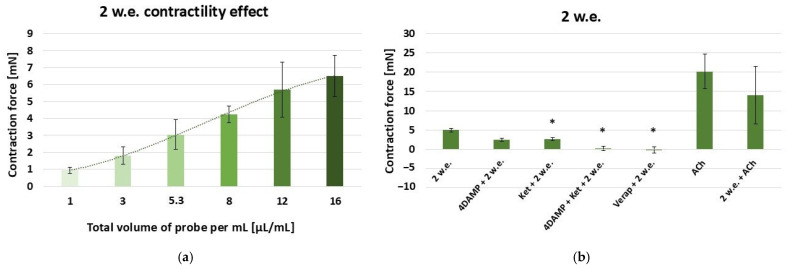
(**a**) A dose-dependent effect of *M. friwaldskyanum* stem extract (2 w.e.) on the contractility of isolated gastric SM samples (n = 6); (**b**) Induced mechanical responses of gastric SM samples (n = 6) by different biologically active substances—alone and in combination with 2 w.e. Effective doses: 2 w.e.—10 µL/mL; 4DAMP—1 × 10^−6^ M; ketanserin—1 × 10^−6^ M; verapamil—1 × 10^−5^ M; Ach—1 × 10^−6^ M. The data is presented as Mean ± SD. The symbol * indicates significant differences versus the control group.

The data on the contractility effects of the water stem extract is presented in Figure 2. Similarly to 1 w.e., 2 w.e. also produced a dose-dependent contractile reaction of the stomach SM in the range: 10–160 µL (effective work dose range: 1–16 µL/mL) [Figure 3a].

The control reaction caused by 10 µL/mL of 2 w.e. (4.97 ± 0.5 mN) as well as the ones induced by the application of 10 µL/mL of 2 w.e. after other biological substances are presented in Figure 3b. When applied after 1 × 10^−6^ M 4DAMP, the aqueous stem extract induced a contraction with force 2.47 ± 0.4 mN, which was not significantly lower than the control one. A significant difference was observed in the reaction provoked by consecutive application of 1 × 10^−6^ M ketanserin and 2 w.e. (2.61 ± 0.36 mN) and the control reaction. However, the mixture containing all three substances (1 × 10^−6^ M 4DAMP, 1 × 10^−6^ M ketanserin and 2 w.e.) was shown to induce a response of 0.1 ± 0.05 mN, which, according to the statistical analysis, was significantly reduced compared to the control reaction (*p* ≤ 0.05). Applied approximately 5 min after verapamil (1 × 10^−5^ M), 2 w.e. led to a relaxation of −0.12 ± 0.8 mN, with the reaction being significantly reduced compared to the control one (*p* ≤ 0.05). ACh in concentration 1 × 10^−6^ M induced a mean contraction of 20.21 ± 4.67 mN, while its combination with 2 w.e. provoked a response of 14.01 ± 7.5 mN. No statisticaly significant difference was observed between the reactions induced by ACh alone and in combination with the extract.

#### 3.3.3. *M. friwaldskyanum* Leaf Extract

**Figure 4:**
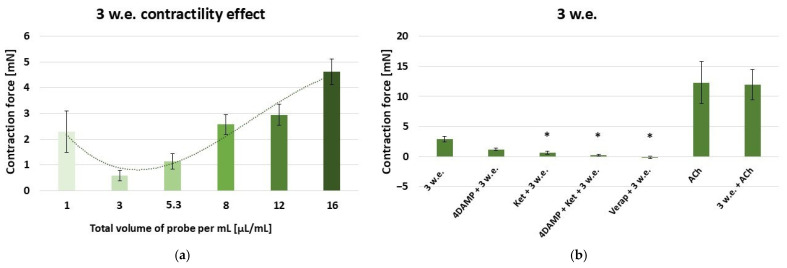
(**a**) A dose-dependent effect of *M. friwaldskyanum* leaf extract (3 w.e.) on the contractility of isolated gastric SM samples (n = 6); (**b**) induced mechanical responses of gastric SM samples (n = 6) by different biologically active substances—alone and in combination with 3 w.e. Effective doses: 3 w.e.—10 µL/mL; 4DAMP—1 × 10^−6^ M; ketanserin—1 × 10^−6^ M; verapamil—1 × 10^−5^ M; Ach—1 × 10^−6^ M. The data is presented as Mean ± SD. The symbol * indicates significant differences versus the control group.

The aqueous leaf extract induced a dose-dependent contractile reaction of the gastric SM in the range: 10–160 µL and effective work dose range: 1–16 µL/mL [Figure 4a].

The following experiments were conducted with 10 µL/mL of 3 w.e., which induced a response of 2.91 ± 0.5 mN [Figure 4b]. The application of the extract post-1 × 10^−6^ M 4DAMP treatment induced a contraction of 1.18 ± 0.19 mN, which was not significantly different to the control reaction. The consecutive application of 1 × 10^−6^ M ketanserin and 3 w.e. demonstrated a significantly reduced response compared to the control (0.65 ± 0.26 mN vs. 2.91 ± 0.5 mN, *p* ≤ 0.05). The mixture of 1 × 10^−6^ M 4DAMP, 1 × 10^−6^ M ketanserin and 3 w.e., induced a contraction with force 0.13 ± 0.07 mN, which was significantly lower than the control one (*p* ≤ 0.05). The application of 3 w.e. after 1 × 10^−5^ M verapamil caused a relaxation reaction of −0.14 ± 0.07 mN. The reaction was significantly lowered in comparison to the control one (*p* ≤ 0.05). ACh in concentration 1 × 10^−6^ M induced a mean contraction of 12.3 ± 3.45 mN. The observed contraction force produced by the combination of 1 × 10^−6^ M ACh and 3 w.e. was 11.93 ± 2.51 mN. No significant difference was observed compared to the sole ACh reaction.

#### 3.3.4. *M. peregrinum* Herb Extract

**Figure 5:**
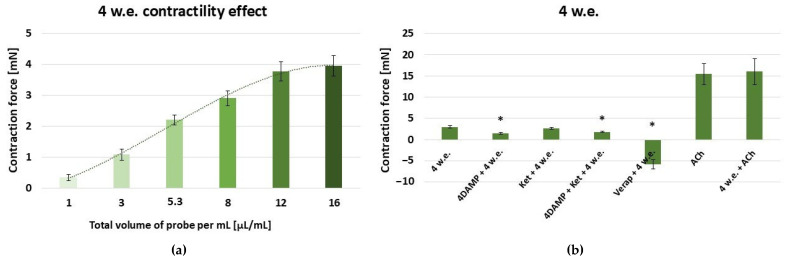
(**a**) A dose-dependent effect of *M. peregrinum* herb extract (4 w.e.) on the contractility of isolated gastric SM samples (n = 6); (**b**) induced mechanical responses of gastric SM samples (n = 6) by different biologically active substances—alone and in combination with 4 w.e. Effective doses: 4 w.e.—10 µL/mL; 4DAMP—1 × 10^−6^ M; ketanserin—1 × 10^−6^ M; verapamil—1 × 10^−5^ M; Ach—1 × 10^−6^ M. The data is presented as Mean ± SD. The symbol * indicates significant differences versus the control group.

As evident form Figure 5a, 4 w.e. induced a dose-dependent contractile reaction in the range: 10–160 µL (effective work dose range: 1–16 µL/mL).

An effective dose of 10 µL/mL was used for the following experiments [Figure 5b]. The *M. peregrinum* herb extract produced a SM contraction of 2.99 ± 0.25 mN. The application of 10 µL/mL of 4 w.e. approximately after 1 × 10^−6^ M 4DAMP led to a significantly reduced response compared to the control group (1.46 ± 0.15 mN vs. 2.99 ± 0.25 mN, *p* ≤ 0.05). Ketanserin (1 × 10^−6^ M) treatment and a following application of 4 w.e. caused a contraction of 2.6 ± 0.20 mN. No statistical significance was observed in comparison to the control group. The combination of 1 × 10^−6^ M 4DAMP, 1 × 10^−6^ M ketanserin, and 4 w.e. induced a response of 1.81 ± 0.12 mN, which was significantly reduced in comparison to the control reaction (*p* ≤ 0.05). Applied post verapamil (1 × 10^−5^ M) treatment, 4 w.e. produced a relaxation reaction of −5.88 ± 1.08 mN, which, according to the statistical analysis, was significantly different to the control one. Treatment of the SM with 1 × 10^−6^ M ACh induced a mean contraction of 15.42 ± 2.48 mN. Similarly, its combination with 4 w.e. caused a response of 16 ± 3.09.

## 4. Discussion

Herbal beverages have a vast popularity as natural remedies and are gaining ongoing recognition as rich sources of bioactive compounds [32]. Furthermore, the interest in medicinal plants and their usage as adjuncts in addition to other treatments has risen in the past years due to increasing toxicities as well as the high cost of conventional therapies [33,34]. Representatives of the *Marrubium* genus, such as *M. vulgare*, have been well investigated and commonly applied for the treatment of various disorders such as digestive and respiratory disorders [35,36]. There are, however, members of the genus which have been less extensively studied and may turn out to be valuable sources of bioactive ingredients that could potentially be used in the treatment of diseases.

The present study focused on the exploration of the antioxidant and antispasmodic activity of water extracts of the aerial parts of two representatives of genus *Marrubium*, namely the endemic *M. friwaldskyanum*, divided into its anatomical parts, and *M. peregrinum* whole herb.

The results of the assessment of the antioxidant content demonstrated the highest abundance of TP and TF in the *M. friwaldskyanum* leaf extract, followed by the *M. peregrinum* herb extract and *M. friwaldskyanum* stem extract, which showed similar concentration of the analyzed compounds. Genus *Marrubium* is known for its affluent phenolic compound content [5]. According to our preliminary study on both plants’ metabolome and more specifically the secondary metabolite analysis, *M. friwaldskyanum* leaves contain numerous antioxidative molecules such as forsythoside D, secologanic acid, isorhamnetin and its derivatives (isorhamnetin-3-O-glucoside and isorhamnetin-3-O-rutinoside). The metabolome profiling also discovered characteristic antioxidants in the stems (salidroside and alyssonoside) and inflorescences (luteoloside, apigenin coumaroylglucoside, hydroxycinnamates and their derivatives) of *M. friwaldskyanum*, as well as in *M. peregrinum* herb (isovitexin, procyadin B, naringenin dihydrochloride) [12]. The lower TP and TF concentration detected in the respective extracts, however, is the likely reason for their less pronounced antioxidant activity compared to the *M. friwaldskyanum* leaves extract.

In consistency with the determined concentration of TP and TF in the extracts, and as evident from the ORAC, HORAC, DPPH, and FRAP assay results, the *M. friwaldskyanum* leaf extract exhibited the most pronounced antioxidant activity, followed by the *M. peregrinum* herb extract and the *M. friwaldskyanum* stem extract. Such a proportional correlation between TP and TF content and the antioxidant activity of *Marrubium* plant extracts has been previously reported [37]. The assessed oxygen radical absorbance capacity of the extracts ranged between 1258.8 µM TE/g DW for the *M. friwaldskyanum* leaf extract and 587.9 µM TE/g DW for the inflorescence one. Almost two times lower peroxyl radical neutralizing ability was observed for the other two extracts compared to the *M. friwaldskyanum* leaf one. Similar results were obtained in the hydroxyl radical averting capacity assay with a range of 356.1–102.9 µM GAE/g DW. The observed antioxidant activity of the *M. friwaldskyanum* leaf extract is comparable to the one recently reported for a *M. vulgare* water extract [38]. The ability of the studied extracts to reduce Fe^3+^ assessed in the FRAP assay varied between 41.27 and 8.08 µM TE/g DW. A significant antioxidant activity of leaf extracts of other *Marrubium* species has been reported with FRAP results similar to ours and the effect was attributed to the antioxidant content of the plants [39,40].

According to the results from the DPPH test, the *M. friwaldskyanum* leaf extract showed the highest % RSA, followed by the *M. peregrinum* herb extract, while the rest of the extracts reduced the DPPH radical to a lower extent. The antioxidant potential of *M. peregrinum* extracts have been reported by other authors [22,23]. Contradictory to our findings for the *M. friwaldskyanum* leaf extract, Kozyra et al. have shown that the in vitro antioxidant capacity of *M. friwaldskyanum* assessed by a DPPH assay was comparatively low [5]. The discrepancy in the results could be explained by the TP content, which in our case turned out to be twice as high, with a possible reason being that the plant we used was collected from its natural habitat.

The other aspect of the current research was the effect of the studied *Marrubium* species on the contractility of isolated rat gastric SM. The results revealed a dose-dependent contractile response to all four extracts. The SM contraction induced by 10 µL/mL of *M. friwaldskyanum* inflorescence extract was inhibited to a similar extent by both 4DAMP and ketanserin. These results imply the involvement of the cholinergic and possibly the 5-HT mediation [41]. Another possible interaction with those pathways may involve an enhanced extract-induced release of ACh and other contractile agents such as 5-HT and/or dopamine in the smooth muscles’ intramural structures [42]. The simultaneous inhibition of the M_3_ and 5-HT_2_ receptors causes a significant reaction reduction, but with residual contraction, which is a possible effect of a third type contractile agent. In combination with verapamil, 1 w.e. provokes a relaxation reaction, which indicates the dominance of intracellular relaxant mechanisms in the absence of Ca^2+^ influx or another Ca^2+^-independent relaxation. The latter could be mediated by the cGMP-dependent protein kinase type I [43]. An alteration of the population of M-cholinoreceptors could explain the tendency towards ACh-induced reaction elevation when combined with the *M. friwaldskyanum* inflorescence extract. Another possible explanation for the increased response to ACh in combination with 1 w.e. is a probable inhibitory effect of the extract on the acetylcholinesterase enzyme.

Similar results were observed for the *M. friwaldskyanum* stem extract, with this difference that its combination with ACh induced a reduced response, although statistically insignificant (*p* ≥ 0.05). The latter effect could be explained by the possible decrease in the number of cholinoreceptors and/or their sensitivity, or by an increase in the acetylcholinesterase activity induced by the contents of the extract.

The effect of the *M. friwaldskyanum* leaf extract on the SM contractility was analogous to that of the other two *M. friwaldskyanum* extracts. The results indicate a more pronounced ketanserin-induced inhibition compared to 4DAMP, possibly because of a stronger effect of the extract on the monoamine mediation, the release of another contractile factor and weaker cholinergic influence compared to the inflorescence and stem extracts.

The *M. peregrinum* herb extract induced a contractile dose-dependent reaction, mediated by SM muscarinic response with a dominant M_3_ activity as shown by the minimized by 4DAMP effect. Ketanserin did not significantly affect the development of the contraction, initiated by 4 w.e. The latter points to a lack of serotoninergic mediation in the mechanism of action of the extract. The simultaneous blocking of M_3_ and 5-HT_2_ receptors decreases the contraction response in a similar magnitude to 4DAMP, which supports the observed results with ketanserin. The observed transformation of the reaction into highly relaxant when 4 w.e. was combined with verapamil suggests a similar mechanism of action to the inflorescence extract.

From the obtained results on the contractility reactions, it can be deduced that a substantial part of the response that the extracts cause has a holinergic character, primarily mediated by M_3_ receptors. The *M. friwaldskyanum* leaf extract makes an exception, as the predominant mechanism of reaction development most probably involves a serotoninergic and/or dopaminergic pathway activation. In addition to the neuromediators mentioned, all extracts seem to activate the release of other contractile and relaxant agents. The blockage of the L-type Ca^2+^ channels transforms the induced effect into relaxation.

Representatives of genus *Marrubium* have long been known to exert spasmolytic activity, although it has been proposed that this effect is tissue specific. Schlemper et al. have demonstrated that a hydroalchoholic *M. vulgare* extract exerted antispasmodic activity in SM preparations of rat uterus and guinea pig ileum but failed to suppress ACh-induced contractions in rat stomach [24]. In our in vitro studies, we did not observe a direct antispasmodic effect of the extracts when applied alone. However, in combination with verapamil such an effect was manifested. Furthermore, an in vitro anticholinesterase activity has been reported for numerous *Marrubium* species [18,44,45]. A recent study on the anticholinesterase potential of eight *Marrubium* taxa revealed that *M. peregrinum* possessed the highest inhibitory activity [46]. Such enzyme inhibitory activity has been shown to correlate with plants’ phenolic content [47].

The discovered stomach prokinetic effect on behalf of both plants could be beneficial in the symptomatic treatment of gastrointestinal conditions such as dyspepsia or flatulence through increasing stomach secretion and motility and accelerating gastric emptying.

## 5. Conclusions

This is the first study to report on the in vitro antioxidant activity and rat gastric SM contractility effect of aqueous extracts from two *Marrubium* species growing on the territory of the Republic of Bulgaria. From the data on the antioxidant activity, we could deduce that out of all four extracts the *M. friwaldskyanum* leaf extract exerts the most significant antioxidant effect, followed by the *M. peregrinum* one. Furthermore, our preliminary assessment of the impact of the extracts on the SM contractility proposes possible involvement of the cholinergic and serotoninergic systems and some calcium dependence of the reactions. Further research is of necessity as for the precise intricate mechanisms of the discovered biological activity of the extracts to be thoroughly elucidated. The obtained results will, however, set the basis for future in vivo analyses through which the therapeutic potential of the plants will be investigated.

## Figures and Tables

**Table 1:** Extraction yield, % DW.

*M. friwaldskyanum* Inflorescence Extract	*M. friwaldskyanum* Stem Extract	*M. friwaldskyanum* Leaf Extract	*M. peregrinum* Herb Extract
24.54	23.46	30.65	27.01

**Table 2:** Total polyphenol and flavonoid content of aqueous extracts of *Marrubium friwaldskyanum* Boiss. and *Marrubium peregrinum* L.

Sample	Total Polyphenol Content, mg GAE/100 g DW	Total Flavonoid Content, mg QE/100 g DW
***M. friwaldskyanum*** **inflorescence extract**	1166.2 ± 37.1 ^c^	181.5 ± 5.4 ^b^
***M. friwaldskyanum*** **stem extract**	1831.6 ± 100.1 ^b^	250.2 ± 7.3 ^b^
***M. friwaldskyanum*** **leaf extract**	3936.0 ± 93.6 ^a^	601.7 ± 5.7 ^a^
***M. peregrinum*** **herb extract**	1855.8 ± 88.4 ^b^	251.8 ± 24.6 ^b^

Results are presented as mean ± SD. Different lower case superscript letters indicate significant differences between the extracts (all *p* values ≤ 0.001).

**Table 3:** Antioxidant activity of aqueous extracts *of Marrubium friwaldskyanum* Boiss. and *Marrubium peregrinum*.

Sample	ORAC, µM TE/g DW	HORAC, µM GAE/g DW	DPPH, %RSA	FRAP, µM TE/g DW
***M. friwaldskyanum*** **Inflorescence Extract**	587.9 ± 16.2 ^b^	129.5 ± 1.7 ^b^	15.17 ± 1.04 ^b^	18.95 ± 0.24 ^b^
***M. friwaldskyanum*** **Stem Extract**	640.1 ± 23.2 ^b^	102.9 ± 0.9 ^b^	15.83 ± 0.60 ^b^	16.42 ± 0.13 ^c^
***M. friwaldskyanum*** **Leaf Extract**	1258.8 ± 53.0 ^a^	356.1 ± 4.7 ^a^	20.28 ± 0.87 ^a,^**^,##^	41.27 ± 0.25 ^a^
***M. peregrinum*** **Herb Extract**	651.0 ± 23.1 ^b^	107.3 ± 2.1 ^b^	19.57 ± 1.60 ^a,^**^,#^	8.08 ± 0.20 ^d^

Results are presented as mean ± SD. Different lower case superscript letters indicate significant differences between the extracts. The symbol ** indicates significant differences versus the *M. friwalsdskyanum* inflorescence extract (1 w.e.) (*p* ≤ 0.01). The symbol ## indicates significant differences versus the *M. friwalsdskyanum* stem extract (2 w.e.) (*p* ≤ 0.01), and # indicates significant differences versus the *M. friwalsdskyanum* stem extract (2 w.e.) (*p* ≤ 0.05); all other *p* values ≤ 0.001. ORAC—oxygen radical absorbance capacity; HORAC—hydroxyl radical averting capacity; DPPH—2,2-diphenyl-1-picrylhydrazyl radical scavenging activity; FRAP—ferric reducing antioxidant power.

## Data Availability

The raw data supporting the conclusions of this article will be made available by the authors on request.
